# Supplementary figures and images for: Cell recruitment and the origins of Anterior-Posterior asymmetries in the Drosophila wing
Source: PLoS One. 2025 Jan 3;20(1):e0313067. doi: 10.1371/journal.pone.0313067 (PMC11698434; doi:10.1371/journal.pone.0313067)

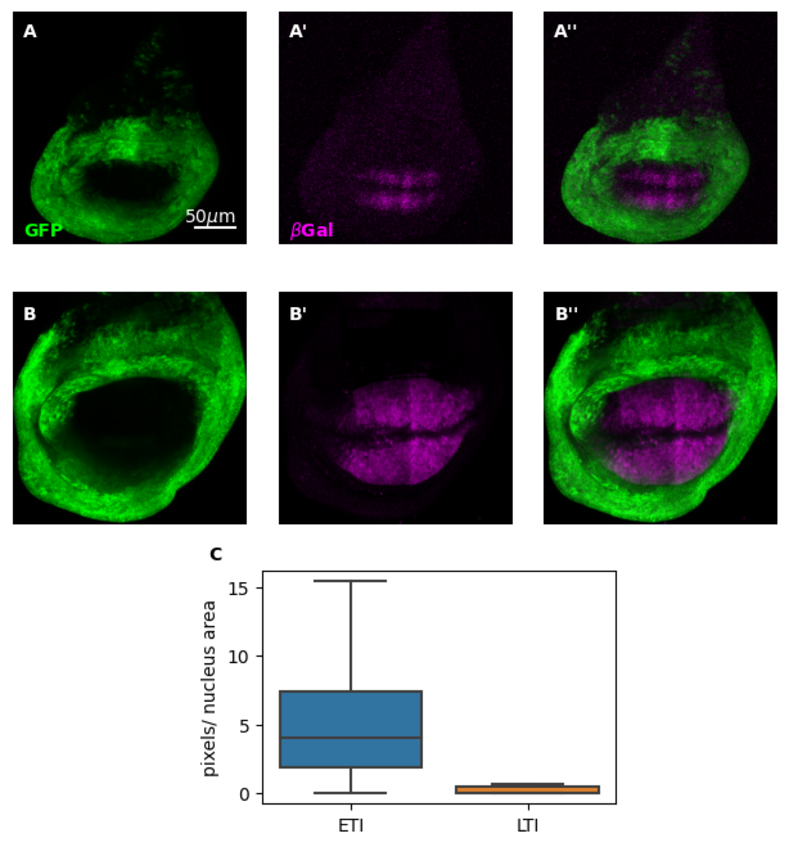

Supplement: S1 Fig — A,B. Control wing disc expressing GFP driven by ds using the Gal4-UAS system during early third instar (ETI) (A) and late third instar (LTI) (B), immunostained with β-galactosidase (A’, B’). A’’ and B’’ show the merge of these patterns. All discs carry the vgQE-LacZ reporter. C. Approximate number of nuclei that simultaneously express vg and ds at each of the stages analyzed (see Materials and methods). Sample size: ETI wing discs (n = 4); LTI wing discs (n = 6). (TIF) [file pone.0313067.s001.tif]

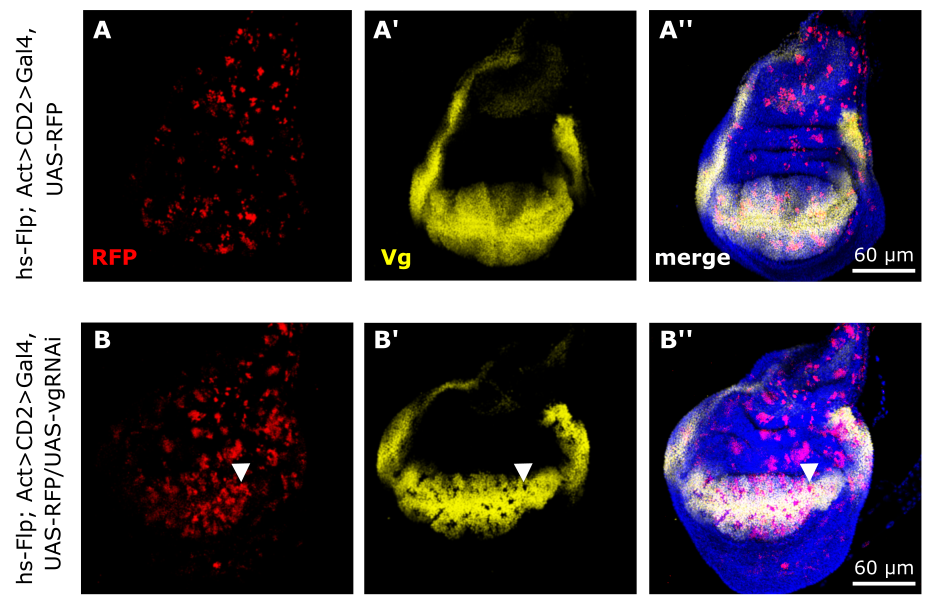

Supplement: S2 Fig — A-B. Generation of genetic mosaics expressing the red fluorescent protein (RFP) through the Gal4-UAS system activated by the FLP-FRT system after heat shock (A) and coexpression of RFP along with vgRNAi (B). A’-B’ Immunostaining with a Vg antibody in the wing disc pouch (A’). Arrowheads indicate a cell nuclei expressing RFP, but lacking Vg expression due to RNA interference action. A’’-B’’ Merge of these patterns. Sample size, n = 9. (TIF) [file pone.0313067.s002.tif]

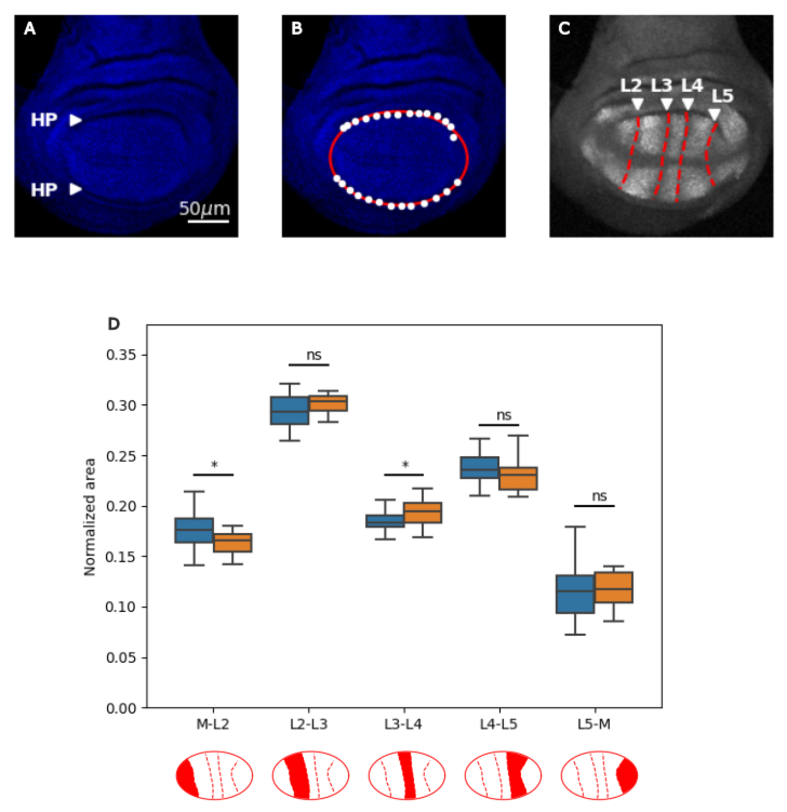

Supplement: S3 Fig — A-C. Representative control wing disc immunostained with DAPI (A,B) and DSRF (C). In A, we mark the position of the HP folds with arrowheads. In B, we mark some points, that pass through the HP fold and were used to fit, by least squares, the ellipse shown in red. In C, the veins are marked with red dashed lines and arrowheads indicate the position of the L2-L5 veins. D. Overlay of the fitted ellipse with the veins to quantify the areas of the intervein sections. The diagrams below this graph illustrate the intervein region analyzed in the wing disc. Sample sizes: control wing disc (n = 30); recruitment-impaired wing disc (n = 16). ns indicates p > 0.05. (TIF) [file pone.0313067.s003.tif]

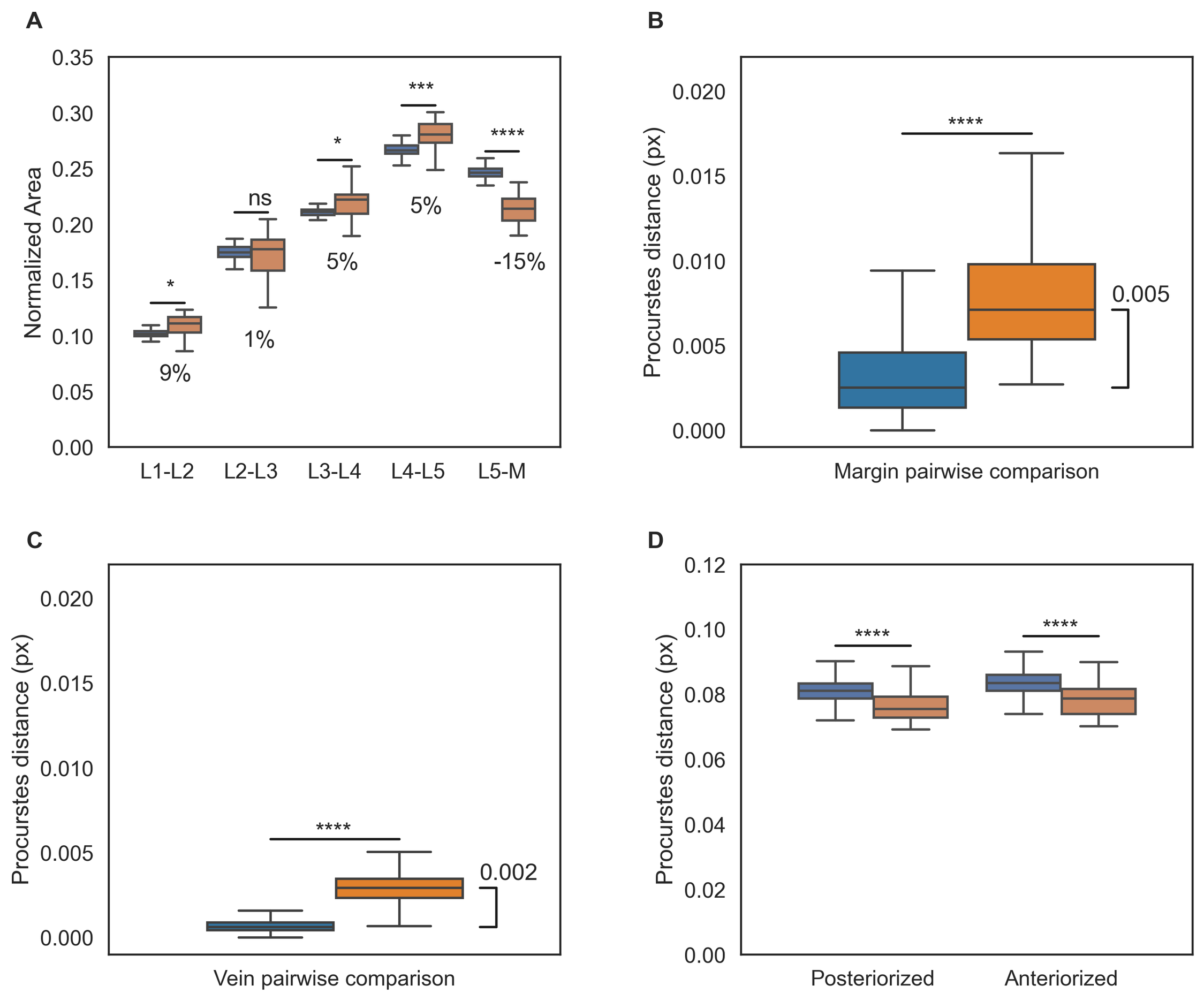

Supplement: S4 Fig — A. Normalized area of each inter-vein region with respect to the total area of the wing. Color coding and analysis is as in Fig 1. B-C. Procrustes comparison of the ML (B) and VL (C). D. Procrustes comparison for Anteriorized and Posteriorized control and recruitment-impaired wings. Color coding and additional information is as in Fig 2. Sample sizes: control wings (n = 58), recruitment-impaired wings (n = 21). A Shapiro test shows that distributions are non-parametric. Thus, a Mann-Whitney U test was conducted. * indicates p < 0.05, *** indicates p < 0.001 and **** indicates p < 0.00005. (TIF) [file pone.0313067.s004.tif]

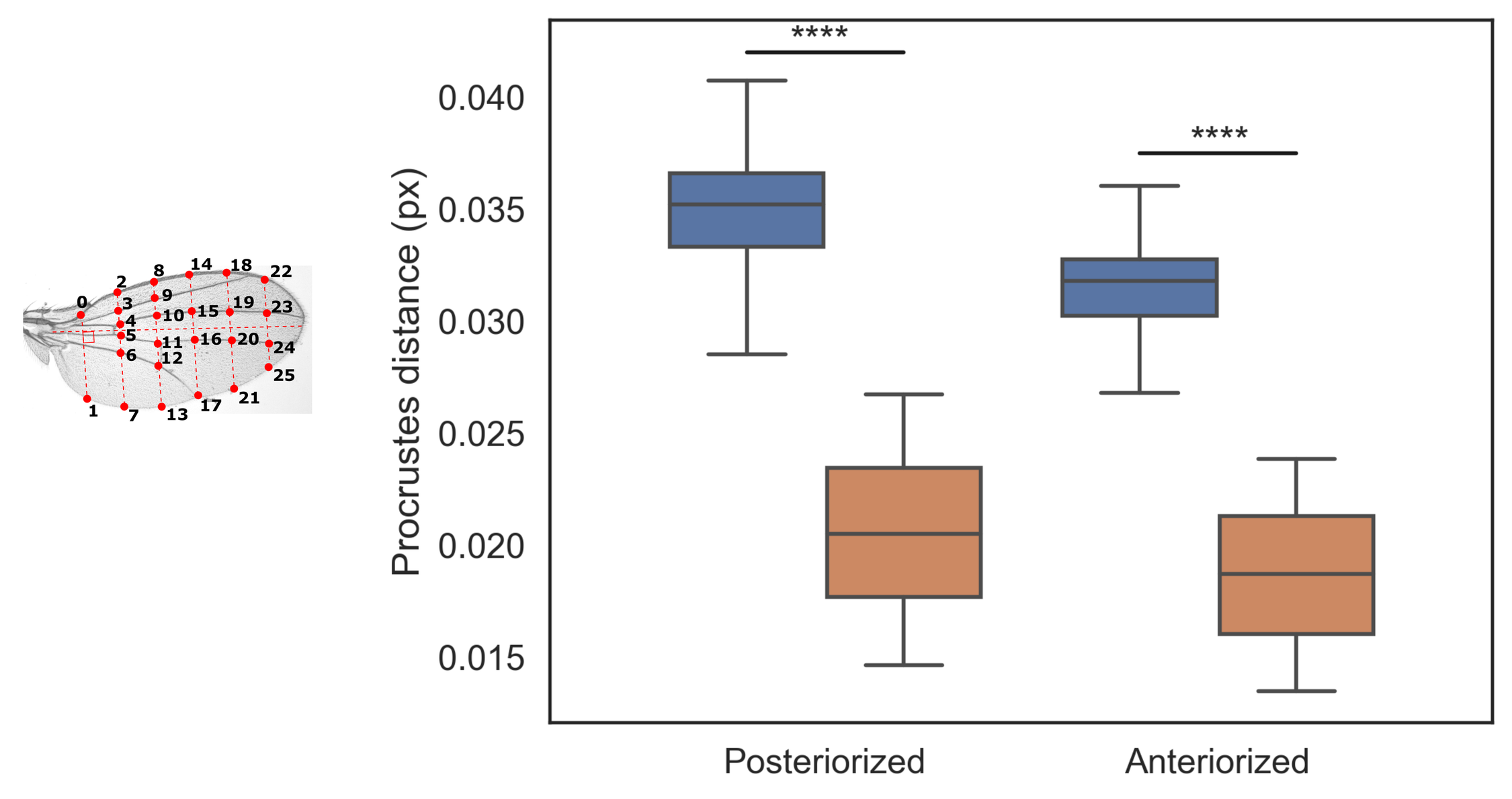

Supplement: S5 Fig — Left. A more comprehensive set of landmarks comprised of 26 points. Right. Procrustes comparison each control and recruitment-impaired wings with respect to their Anteriorized and Posteriorized images using the landmarks shown on the left. Color code is as in Fig 2B. Sample size for each set is as in Fig 2. A Shapiro test shows that distributions are non-parametric. Thus, a Mann-Whitney test was conducted. **** indicates p < 5 × 10^−5^. (TIF) [file pone.0313067.s005.tif]
