# Supplementary material for: Cell recruitment and the origins of Anterior-Posterior asymmetries in the Drosophila wing
Source: PLoS One. 2025 Jan 3;20(1):e0313067. doi: 10.1371/journal.pone.0313067 (PMC11698434; doi:10.1371/journal.pone.0313067)
Supplement: Attachment — Submitted filename: Response to Reviewers.docx [file pone.0313067.s011.docx]

**Detailed response to reviewers’ comments**

Reviewer #1:

Reyes et al. describe an interesting phenomenon that
knockdown of vg results in asymmetric growth of the adult wing in
Drosophila. It shows a great decrease of the L5-M region, whereas other
areas of the wing are increased. However, the authors did not
recapitulate this phenotype in the larval stage. The authors presume
that the overall asymmetry of the adult wing may be attributed to, at
least partially, cell recruitment. However, essential data, such as cell proliferation, is missing to make the conclusion reliable. Below are several comments and criticisms that need to be addressed.

We thank the reviewer for her/his detailed summary of our work. We respond to her/his comments/criticisms below.

1. In Materials and Methods, the authors would clarify:

(1) To show what PEM stands for when it first appears in the text.

We have included a detailed description of PEM in line 109 of the revised manuscript. PEM is a buffer that contains Na-PIPES, EGTA, and MgCl_2_.

(2) How to quantify the relative area?

In wing discs, we used the expression of DSRF to mark pro-veins over the *vg*LacZ expression pattern to define intervein polygons of *vg*LacZ expression. We then computed the relative area by dividing the area of each of these polygons by the total area of the Vg pattern. We also computed relative area in a Vg-independent manner as suggested by another reviewer. This information is presented in lines 124-144 of the revised manuscript.

In adult wings, we measured the area of the intervein sections, obtained as the area of the polygon formed by the veins and/or the wing margin. We then computed the relative area by dividing the area of each of these polygons by the total wing area. This information is presented in lines 171-178 of the revised manuscript.

(3) To show the number of wings for statistical analysis.

Thanks for pointing this out. We have added this information in the revised manuscript. For wing discs, we used n=36 for controls and n=16 for recruitment-impaired samples (line 144). For adult wings, we used n=52 control and n=20 recruitment-impaired adult wings from male flies and n=58 control and n=21 recruitment-impaired adult wings from female flies (lines 177-178). We also reported these sample sizes in the corresponding figure legends.

2. What's the expression pattern of dsGal4 in the wing disc? I hope the authors use only dsGal4 or ds-Gal4 in the whole manuscript.

This is a good point. Sorry that this was unclear. *ds* is expressed in the periphery of the wing pouch*, i.e.*, in cells in cells that have not yet expressed Vg through recruitment. We included this information in lines 99 of the revised manuscript. Also, for consistency, we have replaced *ds*Gal4 with *ds*-Gal4 throughout the text.

3. Label in Fig. 3A or B which line represents L1-L5 and M, respectively, in the larval wing disc.

We have added arrowheads in Fig 3A’’ indicating the pro-vein positions using the DSRF expression pattern. We have also included schemes of the corresponding regions in Fig 3C.

4. The authors found no obvious defects in the L5-M region in the larval stages. What about the area in the pupal stage, such as 32 h after pupation when vein fate induction is conspicuous in the pupal wing (Johannes and Preiss, 2002). What happens to the cell proliferation rate during late third to early pupal stages.

Thanks for this comment. We did not examine changes during pupal stages because the goal of the manuscript is to test the hypothesis of whether or not cell recruitment (which occurs in larval development) contributes to the observed asymmetries in the adult. We do not rule out that changes occur during pupal development, but this is beyond the scope of this manuscript. However, we do include in the Discussion the possibility that changes in the L5-M region arise in early pupal stages (lines 403-406).

5. In Matsuda et al. (2021) work, "it has been shown that the range of the Dpp signal is not required for the growth of the compartment beyond L5", but there are other works to show that the Dpp signaling promotes cell proliferation in the lateral region of the wing disc (Zhang et al. 2013, Development 140, 2917-2922). The authors should reorganize this conclusion.

Thank you for pointing this out. We have added this new information in the introduction (lines 47) stating that the role of Dpp in establishing AP asymmetries remains controversial.

6. To discuss the relevance of more circular phenomenon as shown in Fig.
4 with the reduction of L5-M area, and the increase of other areas?

We agree with the reviewer that this is an aspect that requires further discussion. We have updated lines 406-410 in the Discussion section to provide a simple geometric explanation. Namely, that making an ellipse more rounded implies a reduction in the difference between its major and minor axes, which could explain a reduction at the more lateral areas (L5-M region) while making more central areas larger (L3-L4 and L4-L5; Fig 1B).

Reviewer #2:

In this manuscript, Rosalío Reyes et. al. attempted to figure out the roles of "Vg-cell recruitment", a non-cell-autonomous autoregulation of Vg in the global shaping of Drosophila wing. The authors have previously established the ds-Gal4 > UAS-vg RNAi system which impaired the cell recruitment and found cell-recruitment is
required for the wing to be a complete size. Here, through this "recruitment-impairment fly" the authors showed that cell recruitment is required for the A-P asymmetric shaping of wing. The concept that the cell recruitment did not only regulate tissue growth but tissue shaping is very interesting. However, several interpretations in the experiments do not seem to be logical. I consider that several additional experiments are needed to confirm the authors' argument.

We appreciate that the reviewer finds our hypothesis interesting. We respond to the reviewers’ comments and criticisms below.

Major comments

1. Flies with vg RNAi always have MKRS chromosome in their background
while control flies do not have it. I concern that Minute mutation
(M(3)76A) included MKRS may affect the wing development. The authors
should remove MKRS.

Thank you for raising this technical criticism. It is true that we should have removed the MKRS balancer from our control stock, as it could potentially affect our results. To address this concern, we performed additional experiments that show that the MKRS balancer only has a minimal effect on normalized areas in the adult wing (see graph below). Although significant, the effect of the MKRS balancer in some normalized wing areas does not affect our conclusions. For instance, we observe a significant 3% reduction in the L5-M area in control flies with the MKRS with respect to flies without it, but this reduction is not comparable with the 15% observed in recruitment-impaired flies.

2. The photos showing complementary expression of the ds-Gal4 and vg
expression (vg-lacZ) in the developing wings are needed (e.g. L2, L3 and
wandering L3 stage). I concern that geometrical changing of wing pouch
may not be due to the cell-recruitment impairment but the local
suppression of vg shaped by the unexpectedly overlapped-ds-Gal4 pattern
in the distal region of the wing pouch.

The use of *ds*-Gal4, UAS-*vg*RNAi to impair recruitment is based on the fact that that *ds* expression is transcriptionally inhibited by Vg expression (Zecca and Struhl, 2010), so the Vg and ds-Gal4-driven patterns are not expected to overlap. In any case, we have performed additional experiments to show that this is indeed the case. This is shown in S1 Fig and we further clarify this in lines 98-100 of the Materials and Methods, as well as in lines 243-244 of the Results section of the revised manuscript.

3. (Fig. 1D) The authors should specify the landmarks that exhibit the significant geometrical changing by the cell-recruitment impairment.

Thank you for this valuable suggestion. We added panels E and F in Fig 1 of the revised manuscript to show the specific landmarks that exhibit the most significant differences. The two more significant landmarks are the ones shown with asterisks in Fig 1E.

4.(line 183, Fig. 2B) The authors should explain or discuss what causes the wing shape symmetrically in the recruitment impairment fly (e.g. The XX landmark was shifted to the more posterior/anterior/distal/proximal position). The logical explanation which links Fig1 to Fig2 would make the story clear.

This comment is related to the previous one. In Fig 1E, we show one particular landmark located in the proximal posterior margin (marked with an asterisk) is displaced in a direction orthogonal to the margin and it likely explains the reduction of the L5-M area in recruitment-impaired wings. A description of this observation is included in lines 284-287.

5. (line 100, line 193, Fig. 3) The authors seem to measure each area based on the DSRF expression. However, DSRF would be affected by vg knockdown, thereby affecting the measurement. DSRF staining should be used only for detecting wing veins and the outline of wing pouch should be detected by the fold structure surrounding pouch (e.g. using DAPI staining discs). This may resolve the inconsistency of the authors’ data.

Thank you for this comment. We did use the DSRF antibody to mark the pro-veins in the wing discs. We have updated the subsection of “Analysis of fluorescence patterns in wing discs” in the Materials and Methods section of the revised manuscript to clarify how we determine the intervein territories based on the boundaries defined by the DSRF and the *vg*LacZ pattern (lines 125-134). In our revised manuscript, we also include a new supplemental figure (S3 Fig) to measure intervein areas as suggested by the reviewer (see lines 136-143 and 326-330). Namely, by defining the ellipse outlined by the Hinge-Pouch folds (using DAPI staining) and the pro-veins defined by the DSRF pattern to compute each intervein areas. We did not find significant differences in the relative size of the L5-M section between control and recruitment-impaired discs in either method of measurement.

6. The authors did not show the clear evidence that an asymmetric cell recruitment actually occurs. G-trace system may be helpful for this issue because G-trace system (vg-Gal4 > G-trace) visualizes current and lineage expression of vg, thereby visualizing the expansion of vg provably induced by autoregulation of vg through cell recruitment.

Thank you for this valuable suggestion. We agree that it would be useful to provide evidence of asymmetric cell recruitment. We have not used the G-trace system, but we have used an equivalent lineage tracing system though the expression of a dual fluorescence marker known as TransTimer (He et al., 2009). Using this lineage tracing system, we detected cell recruitment *in vivo* for the first time (Muñoz-Nava et al. 2020), but we did not see differences in AP asymmetries when analyzing these discs, which is consistent with our result of Fig. 3 that no apparent differences could be observed during larval stages.

Minor comments

7. The authors should more carefully explain the concept of cell recruitment in the wing and the ds-Gal4 > UAS-vg.IR system (e.g. The regulation of ds by Vg; short-range induction of vg by adjacent Vg cells, complementary expression of ds and vg, etc.).

Thank you. We have added a more detailed description of cell recruitment and the *ds*-Gal4, UAS-*vg*RNAi system in the Introduction and Materials and Methods sections of our revised manuscript (lines 67-76 and 98-100).

8. (Fig 2B, legend) Are the blue and the orange bars the data values for “control wing vs control-posteriorized/anteriorized wing” and “recruitment-impairment wing vs recruitment-impairment anteriorized/posteriorized wing,” respectively? The authors should describe them carefully as in Fig.1.

We have updated the legend of Fig 2 to clarify this. Thank you.

Reviewer #3:

The manuscript by Reyes et al. described the potential role
of cell recruitment in the establishment of asymmetry during fly wing
development. The authors showed that RNAi knock-down of the selector
gene vg resulted in reduction in adult wing asymmetry. Interestingly,
the authors found that the reduction of the posterior region was not
evident in the larval wing discs. The results are interesting and could
partly support the main conclusion. The manuscript is presented in an
intelligible fashion and well written. I have a few concerns that the
authors should address:

We appreciate that the reviewer’s feedback and his/her appreciation of our work. We respond to the reviewers’ comments below.

Major:
1. How to explain the discrepancy about the phenotypes observed in the
wing disc and the adult wing? If wing discs in later developmental
stages, such as late third instar larvae or early pupal stage were
dissected, could reduction of the L5-M region be observed?

Thanks for these questions. We do not have a clear explanation for this discrepancy and recognize that this is something that needs to be investigated further. Previous work from our lab suggests that cell recruitment occurs during the last half of the third larval instar (Muñoz-Nava et al. 2020). Therefore, our aim was to test the hypothesis that a reduction of the L5-M observed in recruitment-impaired adult wings could be explained by a similar reduction in the corresponding domain in the larvae. We did not find a difference, but we did see a change in the geometry of the pattern (to our knowledge, this is the first time that the shape of a developmental pattern contributes to a symmetry-breaking phenomenon in an adult organ). In our revised manuscript, we added alternative mechanisms in the Discussion section (lines 402-410), including mechanical events occurring during pupal stages.

2. RNAi lines may vary a lot in terms of the knock-down efficiency. Do
other vg RNAi lines produce the same phenotype in the adult wing and
wing discs?

We used an RNAi line from the Vienna Drosophila Stock Centre that has a very clear effect in reducing wing fate differentiation. We did not examine other RNAi lines, but we included a new supplementary figure showing that the effect of this RNAi line is sufficiently strong to reduce Vg levels and function (S2 Fig). We also include this explanation in the Materials and Methods section (lines 101-105).

Minor:

1. In the key words, Drosophila should also be italic.

Fixed, thank you.

2. In the title of Figure 3's legend, " can not recapitulated" should be
"can not be recapitulated".

Fixed, thank you.

Reviewer #4:

The manuscript entitled "Cell recruitment and the origin of
Anterior-Posterior asymmetry in the Drosophila wing" described the
findings that inhibiting vg gene expression during fly wing development
led to defects in wing symmetry. The authors argued that cell
recruitment mediated by vg contributes to the asymmetric shape of the
fly wing. The results are interesting but it is lack of sufficient
evidence to support the conclusion. Some major concerns are listed
below.

We appreciate that the reviewer finds our results interesting. We respond below to the reviewers’ concerns about the evidence supporting our conclusions.

1. The conclusion is based solely on vg RNAi phenotype in the adult
wing. The authors should use RNAi or mutants of other key factors/genes
to disrupt cell recruitment and examine how the wing asymmetry is
affected.

We used *ds*-Gal4, UAS-*vg*RNAi flies since this has been previously demonstrated that it blocks the effects of cell recruitment (Muñoz-Nava *et al.,* 2020). In addition, we now incorporate a supplementary figure that shows that the *vg*RNAi line is very efficient in blocking Vg expression and function (S2 Fig). While we appreciate this suggestion and we agree that it is always better to do more, we believe that in this case the experiments performed are enough to support our conclusion.

2. The authors need to explain more clearly why different conclusions
were made when they and Munoz-Nava et al. used RNAi to knock-down vg
and compared the changes of wing shape?

Muñoz-Nava *et al.,* (2020) blocked cell recruitment using the same knock-down systems, but they focused on the dynamics of the Vg pattern and its contribution to wing growth. They did not examine the effects on wing shape at all. Therefore, our work focuses on other aspects of recruitment (namely, on its contributions to wing shape and AP asymmetries), but there are no conflicting results between the results of Muñoz-Nava *et al.,* 2020 and our work. We clarified this in the Introduction section (lines 76-79) of the revised manuscript.

Reviewer #5:

Firstly, I congratulate you on your work and results. But
some of the details presented in the manuscript are very hard to follow.
Some of the details needed to be addressed before the final publication
of this manuscript.

We thank the reviewer for congratulating us for our manuscript and results, and apologize that he/she found some details hard to follow. We improved our manuscript taking his/her comments as we indicate below.

1. Was any method employed for testing the validity of genetically.
crossed flies?

We used flies that were validated and checked in a recent work from the lab (Muñoz-Nava *et al.*, 2020). However, in order to address this and other reviewers’ concerns, we are including two new supplementary figures in the revised manuscript. S1 Fig shows that the *ds*-Gal4 stock drives expression of a reporter in the desired pattern and S2 Fig shows that the *vg*RNAi line is efficient to down-regulate Vg expression and function.

2. Normal D. melanogaster fly could be used as a control group. What is
the purpose of using genetically modified flies as a control group?

The more adequate control for our experiments would be exactly the same genetic background as the recruitment-impaired genotype, but without the UAS-*vg*RNAi element and this is what we used (with the exception of the MKRS balancer, see our response to Major Comment #1 of Reviewer #2).

3.	What are the purposes of food-restricted experiments? These details are not much discussed in the results and discussion section of this manuscript.

We originally wanted to show that not all reductions in wing size lead to changes in shape and or the proportions of the intervein regions (as in the case of caloric restriction). However, we realized that this was confusing and departed from the story of the paper, so we decided to remove it from the revised manuscript. This of course does not affect any of the original or current conclusions of the work.

4. In the case of immunostaining, is it a novel method or a modification of a previously described method? Try to incorporate references if it is a previously described method.

This is a very standard methodology used widely in this type of research. The protocol was originally obtained from Ethan Bier’s lab at UCSD. We added a sentence in the Materials and Methods section of the revised manuscript to state this (lines 110-111).

5. In the wing area analysis, sample size is missing. However, in the results section, the authors mentioned the sample size, which is really heterogeneous in nature; a homogenous sample size is required for the comparative analysis.

Thank you for this comment. We have now added sample sizes in the Materials and Methods and in all relevant Figure legends. Briefly, we clarify and justify our statistical approach here: First, we determined the normality of the data using a Shapiro-Wilk test. Based on this, we decided to conduct a t-test if both groups had a normal distribution. Prior to this t-test, we verified the equality of variances to decide whether to apply a t-test with equal or different variances. If one of the groups did not have a normal distribution, we applied the non-parametric Mann-Whitney U test, which does not require equal variances. We understand that unequal sample size can be a concern in statistical analysis. However, the tests we chose are robust and may be used for unequal sample sizes (t-test and Mann-Whitney U). Nevertheless, we have added a power analysis to our study to ensure that our tests are sensitive enough to detect significant effects (see the supplementary files for each figure). In all cases, statistical tests show significant differences and the power is greater than 0.95, demonstrating the robustness of our results. We have added these details in the new Statistical Analysis subsection of the Materials and Methods in the revised manuscript (lines 223-238).

6. Geometric morphometric analysis and different multivariate statistical analysis could be used to validate the hypothesis which was discussed in the manuscript. However, the author only conducted preliminary shape changes, why?

Thank you for this comment. We agree that several multivariate statistical analyses can be done for wing shape analysis. We opted for the Procrustes analysis, which is pretty standard and allowed us to compute the average Procrustes distance, which is easy to compute and understand. This methodology has the additional advantage of allowing us to interpret which landmarks are the most important for shape changes (as we now show in Fig 1E,F of the revised manuscript).

7. Sample size is very important for shape analysis. Try to include sample size, both right and left wings used for the analysis? Any separate analysis is conducted for the right and left asymmetry analyses?

Thank you for these questions and suggestions. We clearly specified sample size in the Materials and Methods section of the revised manuscript (lines 144, 177, 194) as well as in each corresponding figure legend. We did not separate right and left wings in the first place but this should not have any impact on AP asymmetries.

8. A separate section for statistical analysis can be added to the methodology section.

Thank you for this suggestion. We have added a new Statistical Analysis subsection in the Materials and Methods section of the revised manuscript.

9. What is the purpose of margin-wise and vein-pairwise comparisons? These details are not much explained in the methodology section.

The adult wing in *Drosophila* has two morphological features, the venation pattern and the shape of the wing blade. We conducted comparisons of landmarks on the margin as well as on the veins to determine which features of the wing are affected the most when cell recruitment is blocked. As we show in the revised manuscript, the shape of the wing margin is affected 2.5 times more than the venation pattern (Fig 1C-F), suggesting that shape of the wing blade is perturbed, while the relative location of the veins is somehow robust when recruitment is impaired (lines 274-282).

10. For the comparative analysis, homogenous landmark selection is very important; the significant venation variations also exhibited by genetically modified flies, how the authors arrived at the selection of homogenous landmarks.

Thank you for this comment. We agree that the selection of landmarks is crucial in this analysis. The selection of landmarks is described in detail in the second paragraph of the Procrustes analysis subsection within the Materials and Methods section. Briefly, the landmarks were selected using representative and reproducible features in the adult wing, such as vein-crossvein intersections and the intersections of veins with the wing margin. These landmarks are easily identified in both control and recruitment-impaired wings.

11. This manuscript had a separate section for results and discussion.
Some of the data are discussed in the results section with proper
citations; this kind of writing is normally used in the discussion
section, not the results section. The results section only explained the
major findings without any comparison with previous findings with
references.

Thank you for this suggestion. In the revised version, we avoided discussing the data within the Results section and left the interpretation to the Discussion section.

12. Based on the previous geometric morphometric analysis, Drosophila
wings exhibit a proximal-distal two modular (i.e., modularity)
compartmentalization; there is no significant modular organization in
AP. Previously, several research articles explained the genetic
mechanisms behind wing development and their modular organizations. Try
to compare these kinds of findings in the manuscript; it will highlight
the novel findings discussed in the manuscript.

Thank you very much for this suggestion; we were not aware of this result. We think it interesting that there is no modular organization along the AP axis, as it supports our idea that AP organization is directly affected by cell recruitment. We have mentioned this in lines 376-379 of the Discussion section in the revised manuscript.

Reviewer #6:

The study addresses an important question in biology: how
the shape of organs is generated and, more specifically, how asymmetries
are formed. The authors use the shape and pattern of the Drosophila wing
as a model. The authors focus on the antero-posterior (AP) asymmetries,
noting that despite symmetric growth signals the wing exhibits a clear
AP asymmetry manifested in the position of the veins. The wing primordia
cells are specified by Vestigial (Vg), whose expression is controlled by
two separate cis-regulatory elements: the vg boundary element (vgBE),
which responds to Notch signaling and activates Vg expression at the
dorsal-ventral (DV) boundary, and the vg quadrant enhancer (vgQE), which
is activated by Wingless (Wg) and Decapentaplegic (Dpp) signaling in the
rest of the wing pouch. Importantly, the vgQE is involved in a cell
recruitment process that depends on a feed-forward signal sent by
Vg-expressing cells to non-expressing cells.

Previous work by the group utilized a genetic system to downregulate
cell recruitment by reducing vg expression in hinge cells (ds>vg-RNAi),
resulting in smaller, but well-proportioned adult wings. The authors
build upon these observations, using quantitative analysis to find that
the vg-recruitment-dependent process contributes to generating the
asymmetry of adult wing flies, specifically at the L5-M domain. To
investigate how cell recruitment in the L5-M region contributes to
breaking the symmetry in the wing, they analyzed markers of intervein
(DSRF) and vgQE to measure the relative area of each intervein region in
larval stages. They conclude that although cell recruitment doesn't
affect the intervein L5-M area in larval discs, this process influences
the circularity of the wing pouch and therefore the shape of the adult
wing.

The paper is well-written, the logic of the manuscript and their
presentation is adequate, and the conclusions are supported by their
results. However, I think to properly understand their results and
conclusions the authors should introduce better the model of cell
recruitment and how they interfere genetically with it.

I have several questions and suggestions that the authors may want to
consider before publication.

We thank the reviewer for the detailed summary of our manuscript and for his/her compliments on our work. We appreciate that the reviewer found the paper adequately presented and supportive of the conclusions. We respond to his questions and suggestions below.

Major comments:

1. The authors should clearly explain in the introduction, results, and methods sections how cell recruitment to the wing pouch region proceeds during development and how they blocked it. The authors rely on their previous paper (Muñoz-Nava et al., 2020); however, in this new work, very little background and experimental design is described. This part must be improved for the readers to understand this new work.

Thank you for this suggestion. We added a more detailed description of cell recruitment in lines 67-76 of the revised manuscript.

2. Are there any other factors besides cell recruitment that could contribute to the wing phenotype of ds>vg-RNAi? As Vg has been implicated in cell survival and proliferation, I am not completely convinced that the entire phenotype could be due to cell recruitment. I think this should be discussed and explained.

This is a very good question for which we do not have a straight answer. In previous work from our laboratory, Muñoz-Nava *et al.*, (2020) showed that upon recruitment-impairment using the same system (*ds-*Gal4, UAS-*vg*RNAi), neither proliferation nor apoptosis are affected. However, it is likely that the *ds-*Gal4, UAS-*vg*RNAi animals has an impact on other aspects such as oriented-cell divisions or tissue mechanics during pupal development as we describe in the Discussion section of the revised manuscript (lines 401-410).

3. Could the authors provide additional data to convince the reader that
the downregulation of Vg is indeed from cells that have been recruited
to the Vg domain? Is the *ds*-Gal4 expressed in cells that have Vg, but
not in those that have been actively recruited? As Ds forms a
proximo-distal gradient of expression, the authors should clearly
demonstrate or at least discuss that in their experiments they are
blocking cell recruitment and no other functions of Vg in survival and
proliferation.

Let us clarify. As we mentioned in comment 1, Muñoz-Nava *et al.,* (2020) showed that upon *vg*RNAi expression in the *ds* domain, there are no effects on cell death or cell proliferation. As we now show in S1 Fig, the patterns of Vg and *ds*-Gal4 do not overlap (there is actually a gap between these patterns suggesting that even graded parts of *ds* do not seem to overlap with Vg either). Therefore, *ds*-Gal4 is actually expressed in cells that are not yet recruited, but will be if they are located within the wing pouch. However, since the *vg*RNAi is expressed in advance of cell recruitment, *ds*-expressing cells cannot express Vg.

Minor Comments:

1. In the introduction (line 50), the authors indicate that “a work by Matsuda et al., (2021) has shown that while anterior patterning and growth require Dpp diffusion, it is dispensable in the posterior compartment.” Please correct me if I am wrong, but the work of Matsuda indicates exactly the opposite, that while critical for posterior patterning and growth, Dpp dispersal is largely dispensable for anterior patterning and growth. Please correct.

Thank you very much for this observation. No, you are not wrong. We have corrected this in our revised manuscript. Please refer to lines 50-52.

2. In Fig. 2 legends and result section, explain better how the comparisons were made between the recruitment-impaired wings and the symmetrized versions.

Thank you for this comment. We have modified the legend of Fig 2 to better explain the comparisons of wings with their symmetrized versions. Additionally, we have added further details in the Procrustes analysis subsection of the Materials and Methods section in the revised manuscript.

3. The analysis of the wing disc to understand how cell recruitment contributes to breaking the AP asymmetry could be improved. First, could the authors indicate in Fig. 3 which regions of the disc contribute to the adult wing?

As we mentioned in the Introduction and new S2 Fig, Vg expression is necessary and sufficient for wing fate determination. Therefore, we use expression of the vgQELacZ reporter to indicate the cells that contribute to the adult wing (the corresponding areas of each intervein areas that presumably contribute to the adult wing is depicted at the bottom of Fig 3C if the revised manuscript.

4.	Line 217: Please correct “invictus” to “invected.”

Fixed, thank you.

Reviewer #7:

This paper investigated the roles of cell recruitment in
establishing tissue asymmetry using Drosophila wing development as a
model. Wing growth relative to the organ's AP axis was analyzed by using
a combination of genetic manipulations and morphometric analyses of wing
phenotypes. The paper shows that the impairment of cell recruitment
causes a more symmetric adult wing relative to the AP boundary. However,
surprisingly, there were local asymmetric patterns of inter-vein growth,
which collectively caused the overall symmetric outcomes. While most of
the inter-vein regions of the wing showed an increase in growth, the
L5-M region of the wing showed a reduction. However, in the larvae, the
impairment of cell recruitment did not reduce the L5-M region of the
wing imaginal disc from where the adult wing was derived. Instead, the
shape of the wing disc pouch was changed. The results suggested some
role of cell recruitment in shaping asymmetry, but the exact mechanisms
remained elusive. Cell recruitment could be one of the many determinants
that redundantly contribute to the overall asymmetric shape of the adult
wing.

The general role of cell recruitment in asymmetric organ shapes is
interesting. I have a few suggestions:

We are thankful that the reviewer found the hypothesis of our work interesting. We respond to her/his suggestions below.

1) The paper was primarily written for the experts in the field. It
would be helpful to provide more descriptions of the various terms and
experiments for general readers. For instance, a brief
definition/description of cell recruitment in the wing disc context
might be more helpful in conceptualizing the experimental results.

Thank you for pointing this out as this was a recurrent comment of other reviewers as well. We have added a detailed description of the cell recruitment mechanism in lines 67-76, and we clarified some aspects of the experiments in the Materials and Methods section of the revised manuscript.

2) Lines 142 and 189:

Although a previous paper has been cited on the genetic method used
here, a brief description of what genes were manipulated and how is
crucial. This would not only enhance the reader's engagement but also
provide a more complete picture of the research.

Thank you for the feedback. We have added more details to the lines you referred to. You can now review these changes starting from line 242, as well as in the Materials and Methods section of the revised manuscript.

3) What is the relationship of cell recruitment with asymmetric cell
division or asymmetric Dpp/Tkv expression/signaling? It would also be
helpful to know how the authors ensured that the genetic condition
impaired only the cell recruitment process, not the cell division or
asymmetric signaling.

This is a very interesting question and this has not been studied in detail. However, we added some extra details about asymmetric AP signaling and cell recruitment briefly in the Introduction section of the revised manuscript (lines 45-53). We did not explore the question in detail as it is beyond the scope of this study. Regarding the second question, it is known from a previous work that cell division is not affected in recruitment-impaired mutants (Muñoz-Nava *et al.*, 2020).

4) Discussion (line 233): The discussion could be more clarified.

Thank you. The Discussion section has been improved in our revised manuscript.

-------------------------
